# A Rare Case of Iatrogenic Inverted Stress Cardiomyopathy

**DOI:** 10.14797/mdcvj.1110

**Published:** 2022-09-29

**Authors:** Parviz-Ali Lotfian, Arun Umesh Mahtani, Seyed Zaidi, Richard Grodman

**Affiliations:** 1 Department of Medicine, Richmond University Medical Center/Mount Sinai, Staten Island, New York, US; 2 Department of Cardiology, SUNY Downstate Medical Center, Brooklyn, New York, US; 3 Department of Cardiology, Richmond University Medical Center/Mount Sinai, Staten Island, New York, US

**Keywords:** takotsubo cardiomyopathy, myocardial disease, stress cardiomyopathy, myocardiopathies, broken heart syndrome

## Abstract

We discuss a case of a 42-year-old female who was admitted for chronic intractable lower back pain requiring elective spinal surgery. Postoperatively, she experienced chest pressure and abdominal pain with a notable elevation in cardiac markers. A cardiac catheterization and left ventriculogram revealed normal coronary arteries and basal anterolateral hypokinesis, with the normal movement of the distal segment of the anterior wall. A rare variant of stress cardiomyopathy was diagnosed.

## Background

A 42-year-old Hispanic female was admitted to the hospital for an elective anterior lumbar interbody fusion of the L5-S1 vertebrae, secondary to L5-S1 disc degeneration, and intractable L5-S1 disc related pain for the past 10 years. On admission, the patient complained of pain that radiated from her lower back to her bilateral knees, with the left lower extremity pain greater than the right. Additionally, she complained of numbness, tingling, and weakness in the bilateral lower extremities with her legs “giving out.” The pain was described as 7/10 on the pain scale. Physical examination was notable for 05/05 grip and strength in the bilateral upper extremities, 05/05 knee flexion and extension in the bilateral lower extremities along with lumbar paraspinal tenderness, pain greater on the left than the right side. Initial vital signs showed a blood pressure of 118/77 mm Hg, a mean arterial pressure (MAP) of 91 mm Hg, a heart rate of 55 beats/minute, oxygen saturation of 100% breathing ambient air, a respiratory rate of 16 cycles/minute, and a temperature of 98.3 F.

The patient underwent the procedure, which took 8 hours, with blood loss reported to be about 500 ccs. On postoperative day (POD) 1, the patient experienced chest pressure as well as abdominal pain at night. Vital signs were measured every hour and were consistent for hypotension: blood pressure was between 80s to 90s mm Hg systolic and 50s to 60s mm Hg diastolic with a MAP less than 65 mm Hg. She also became anuric and her urine output declined to less than 5 cc/hour. Serum troponin I was measured and trended every 5 hours. A 12-lead electrocardiogram (ECG) and echocardiogram were ordered.

Her medical and surgical history was significant for depression, occasional palpitations, a partial hysterectomy, and gastric bypass 2 years prior to relieve the chronic back pain through weight loss.

## Investigations

The initial level of troponin I was recorded as 1.850 ng/mL on the night of POD 1 with subsequent measurements being 2.250 ng/mL, 1.970 ng/mL, 1.630 ng/mL, and 1.440 ng/mL going into POD 2 (normal: < 0.04 ng/mL). B-type natriuretic peptide (BNP) levels were not measured for this case.

**Figure 1:**
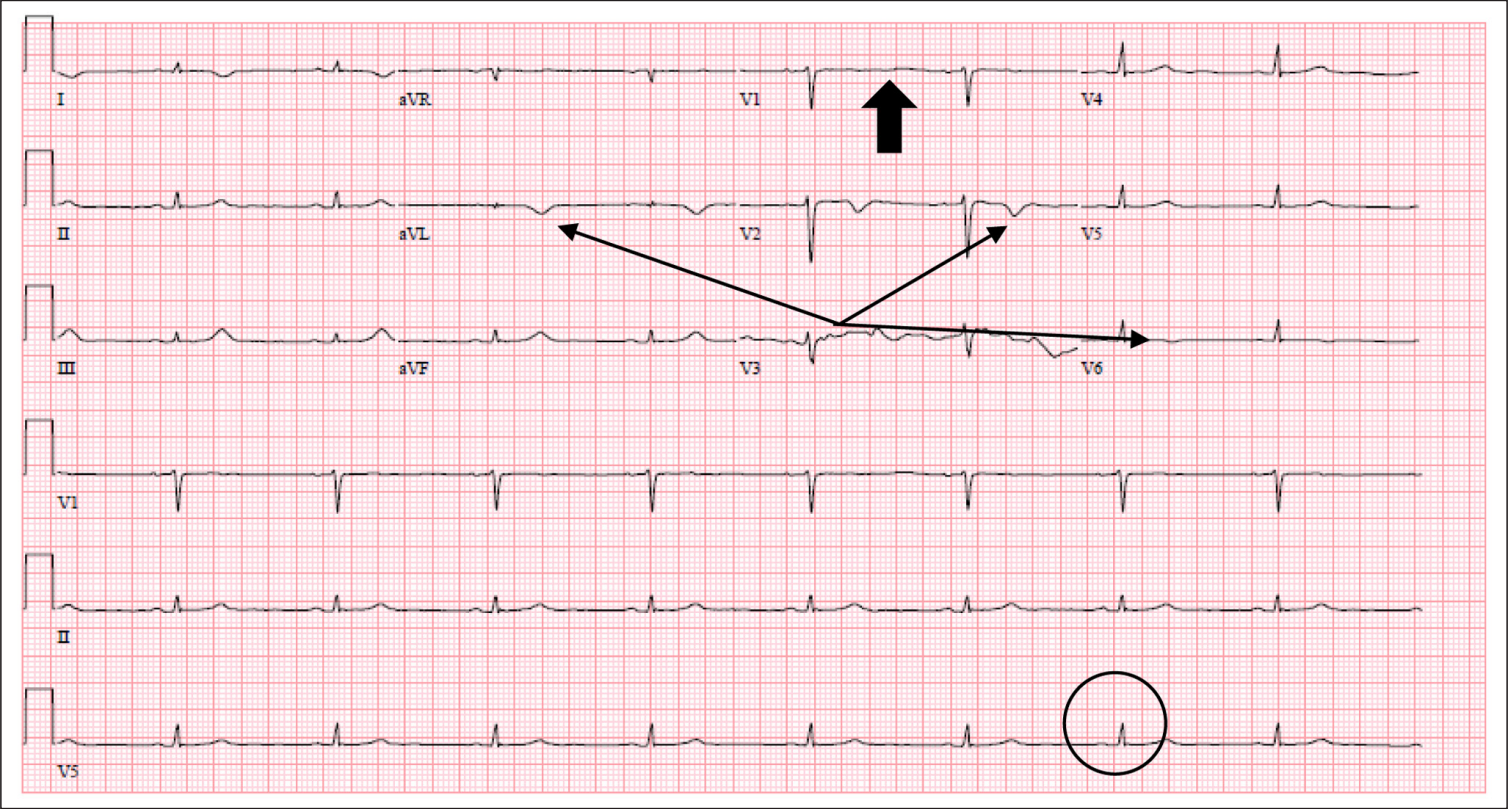
Electrocardiogram on postoperative day 1. Electrocardiogram shows sinus bradycardia with low-voltage QRS complexes (circle), T-wave inversions in leads aVL, V2, V6 (thin arrows), and T-wave flattening in V1(block arrow).

**Figure 2:**
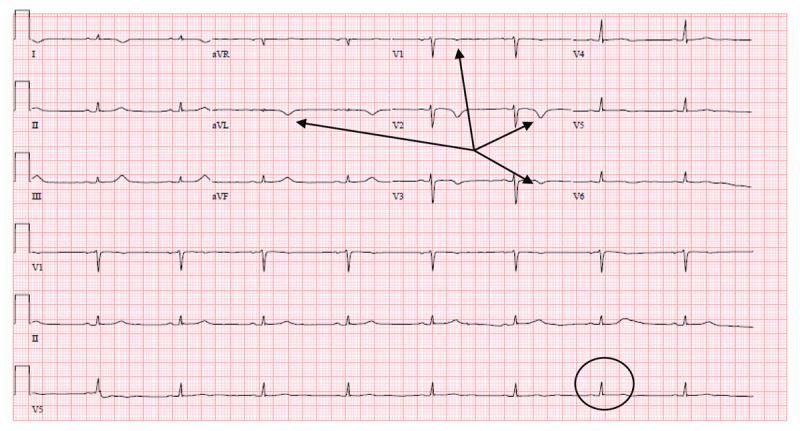
Electrocardiogram on postoperative day 2. Electrocardiogram shows sinus bradycardia with low-voltage QRS complexes (circle), T-wave inversions in leads aVL and V1-V3 (thin arrows).

On the night of POD 1, a 12-lead ECG showed sinus bradycardia with low-voltage QRS complexes, T-wave inversions in leads aVL and V2, and T-wave flattening in V1 (Figure 1). No prior ECG was available for comparison. Repeat ECG on POD 2 showed sinus bradycardia with low-voltage QRS complexes and T-wave inversions in leads aVL and V1-V3 (Figure 2).

An echocardiogram performed on POD 2 showed a normal left ventricular (LV) size, normal global LV systolic function, an LV ejection fraction (LVEF) of 55% to 60%, and hypokinesis of the wall motion of the mid-anterior LV, with mild hypokinesis of the wall motion of the lateral LV. Distal wall motion was normal.

**Figure 3:**
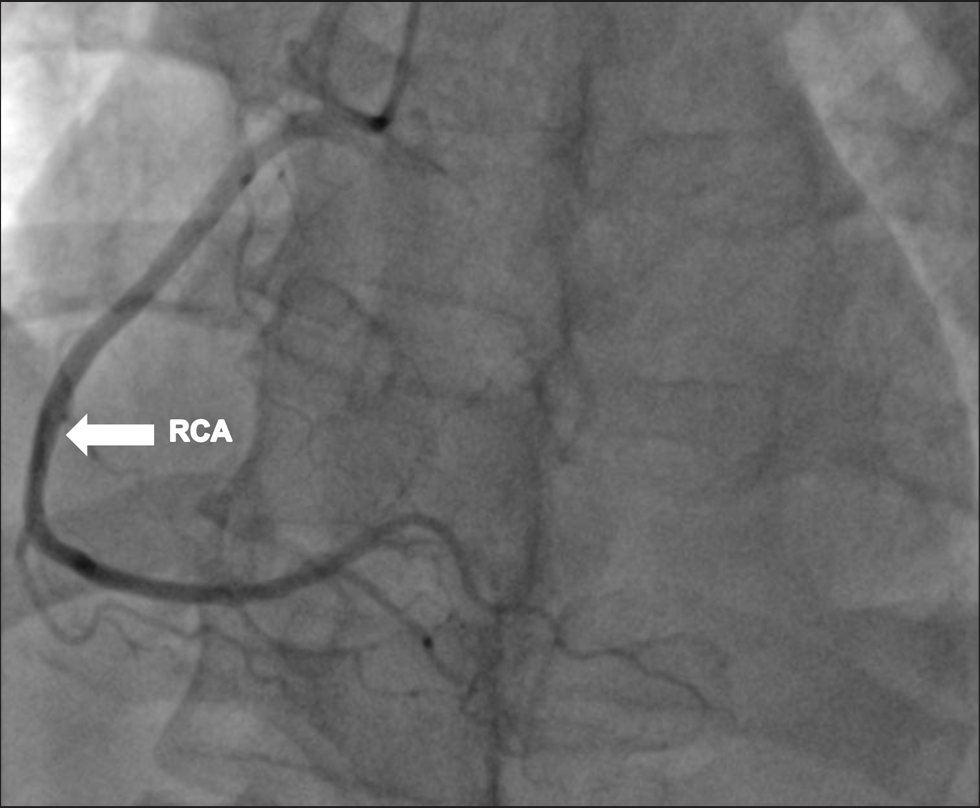
Cardiac catheterization on postoperative day 3. Left anterior oblique (LAO) cranial angulation view shows the right coronary artery along with its branches.

**Figure 4:**
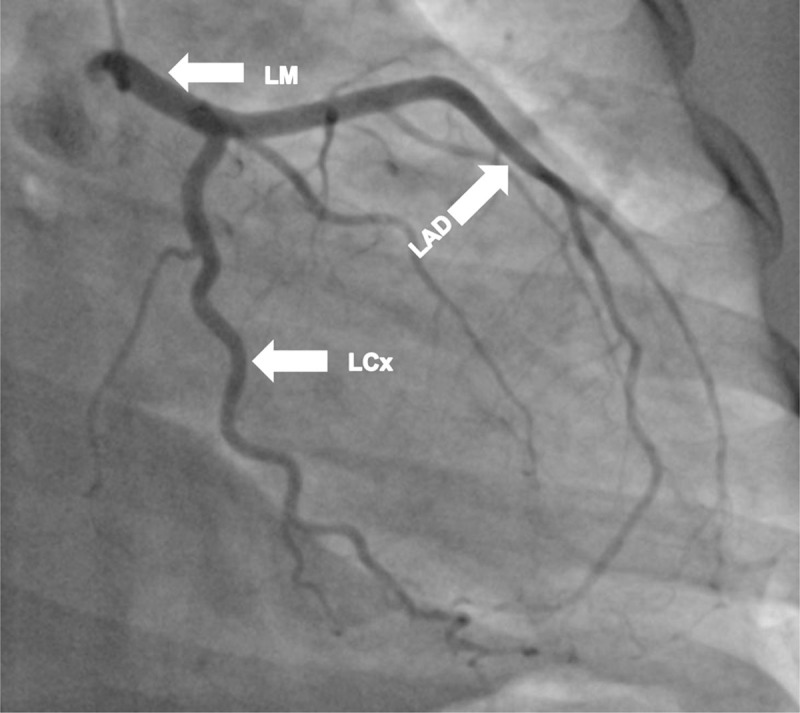
Cardiac catheterization on postoperative day 3. Right anterior oblique (RAO) caudal angulation view shows the left main (LM), left anterior descending (LAD) and left circumflex (LCx) arteries and its branches.

**Figure 5:**
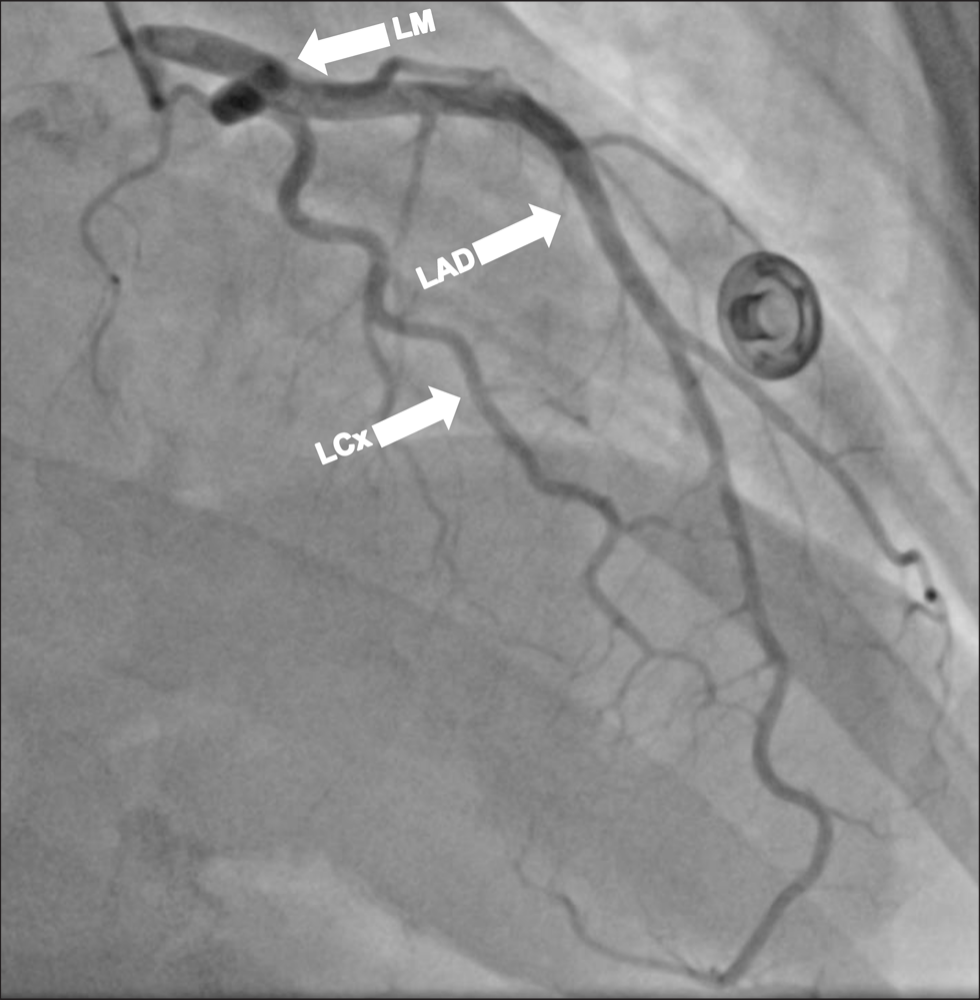
Cardiac catheterization postoperative day 3. Right anterior oblique (RAO) cranial angulation view shows the left main (LM), left anterior descending (LAD), and left circumflex (LCx) arteries along with its branches.

**Figure 6:**
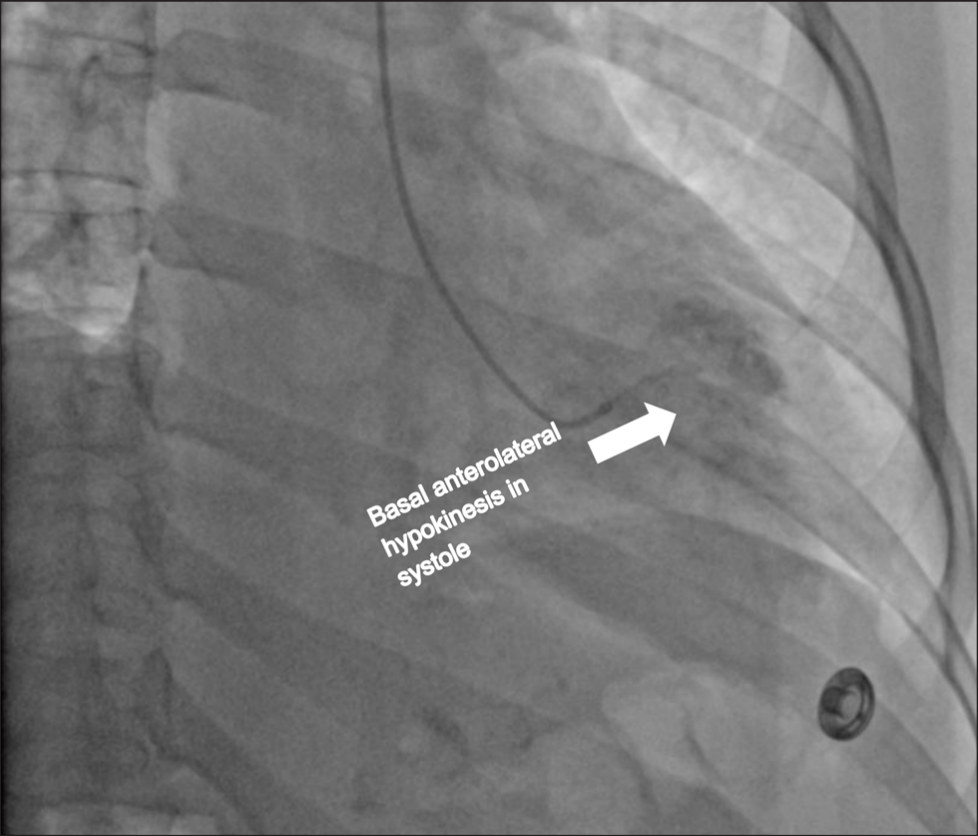
Left ventriculogram on postoperative day 3. Left anterior oblique (LAO) view shows the left ventricle and different segments during systole.

**Figure 7:**
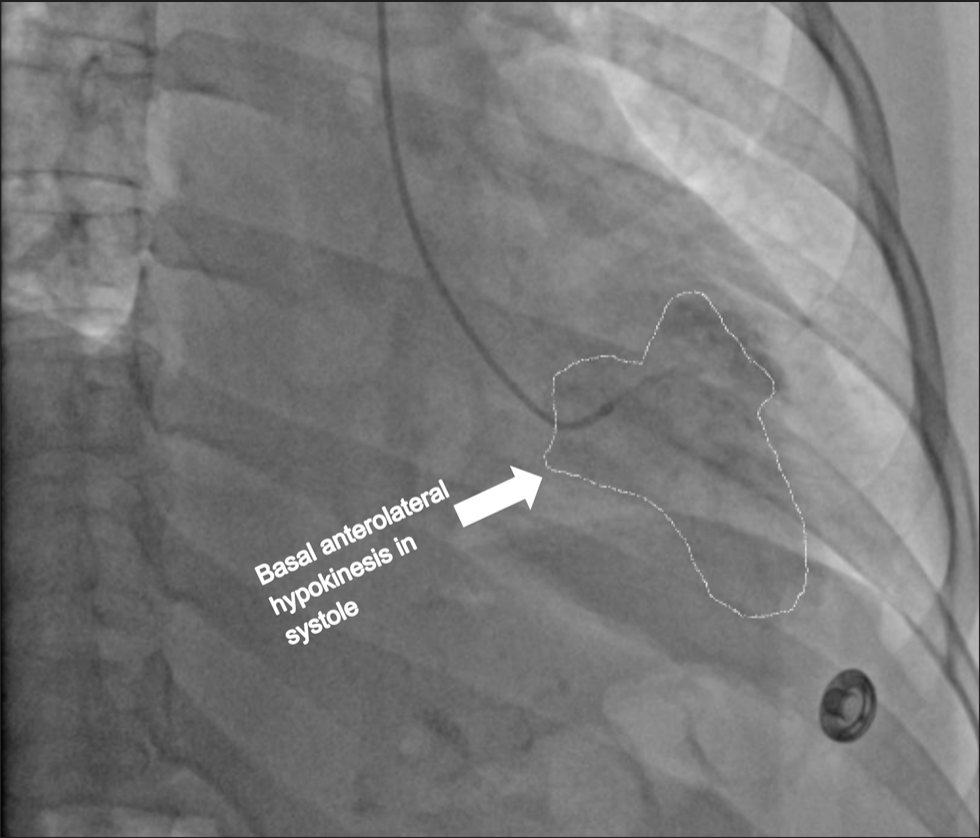
Left ventriculogram on postoperative day 3. Left anterior oblique (LAO) view shows the left ventricle and different segments during systole.

Cardiac catheterization and a left ventriculogram were performed on POD 3. It revealed normal coronary arteries, a grossly normal LV function, and focal anterobasal hypokinesis, with the normal motion of the distal segment of the anterior wall (Figures 3, 4, 5, 6, 7).

Serial troponin I measurements going into POD 4 reported 0.799 ng/mL, 0.733 ng/mL, 0.558 ng/mL, 0.574 ng/mL, 0.479 ng/mL, 0.415 ng/mL, 0.310 ng/mL, and 0.124 ng/mL (normal: < 0.04 ng/mL).

## Differential Diagnosis

Given the investigative findings, our differential diagnosis included (but was not limited to) stress cardiomyopathy, non-ST-elevation myocardial infarction (NSTEMI), pulmonary embolism, acute coronary syndrome, acute aortic dissection, hypovolemic shock, cocaine-related cardiomyopathy, coronary artery vasospasm, and dilated cardiomyopathy.

## Management

The patient was treated with a high-dose statin and anticoagulated with a heparin drip on POD 1, having considered her postoperative status and suspicion of an NSTEMI due to T wave inversions and rise in troponin levels pre-catheterization. There was no neurosurgical contraindication to administering heparin, however aspirin and clopidogrel were avoided. Her vital signs improved with the administration of Lactated Ringer’s, and her troponin levels continued to downtrend. Post-catheterization, the patient had no chest pain or shortness of breath. Heparin drip and high-dose statin were discontinued after she displayed normal coronary arteries on POD 3, and Lactated Ringer’s was discontinued on POD 4. No beta-blockers or angiotensin-converting enzyme (ACE) inhibitors were given due to bradycardia on ECG and low blood pressure readings. Risk factor modification was recommended with follow-up in the cardiology clinic on an outpatient basis.

## Discussion

**Figure 8:**
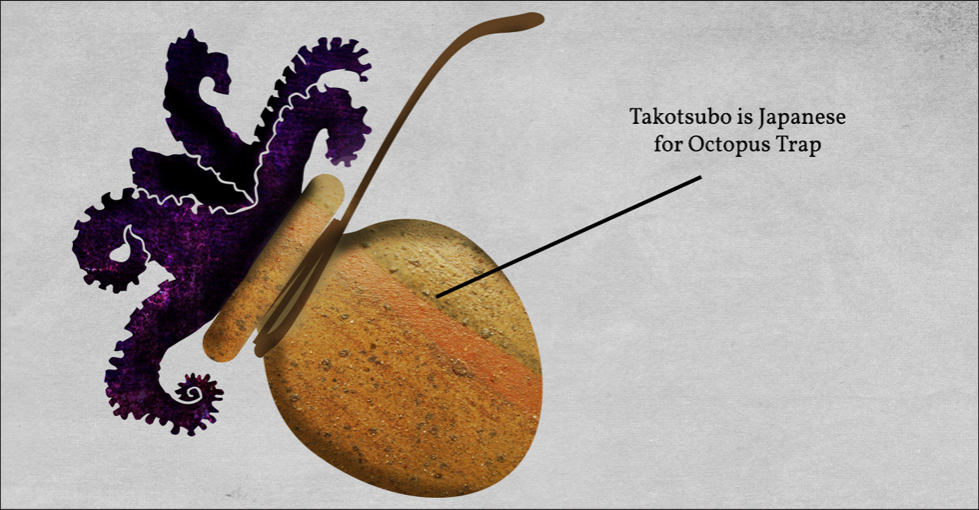
Takotsubo. Source: http://emdidactic.blogspot.com/2016/05/takotsubo-cardiomyopathy.html

Takotsubo cardiomyopathy was first described in the 1990s in Japanese females.^1^ It is also called “stress cardiomyopathy” or “broken heart syndrome.” Stress cardiomyopathy manifests as an apical “ballooning” of the heart, which usually occurs secondary to physical or emotional stress. This ballooning is a result of apical hypokinesis and basal hyperkinesis. LV dysfunction is often reversible and usually resembles an acute coronary syndrome (ACS) with no finding of significant coronary artery disease on an angiogram.^2^ Stress cardiomyopathy received its name due to the heart’s appearance, which is like a Japanese Takotsubo, a pot used to trap octopi (Figure 8).

Multiple variants of stress cardiomyopathy have been described based on which region of the ventricular wall is affected. A rare variant known as “inverted stress cardiomyopathy” presents with basal ballooning, as opposed to the more typical apical ballooning.^3^

The incidence of stress cardiomyopathy is estimated to be approximately 2% of all troponin-positive patients. The incidence of the variant in all stress cardiomyopathy patients is reportedly variable, ranging from 1% to 23%.^4^ In a study from the International Takotsubo Registry, 2.2% of patients were found to have the inverted variant.^5^ While stress cardiomyopathy has been found to occur more frequently in postmenopausal women, the inverted variant has been observed mostly in younger women.

The exact mechanism of injury of stress and inverted stress cardiomyopathy is not well understood. Various theories have been posited, the primary being catecholamine cardiotoxicity. Catecholamines have been known to have toxic effects on the cardiac myocardium. Some postulated mechanisms for this include persistent activation of calcium channels, membrane damage, and microvascular spasm.^6^ It is thought that adrenoreceptors have a higher density in the apex of the heart in postmenopausal women, whereas they have a higher density at the base of the heart in younger patients.^7^

While emotional and physical stress are the most recognized triggers in stress cardiomyopathy,^8^ there have been documented cases of various associations with inverted stress cardiomyopathy, such as multiple types of intracranial hemorrhages,^9^ general anesthesia for surgical and dental procedures,^10,11^ eating disorders,^12^ neurologic conditions such as multiple sclerosis,^13^ and energy drink consumption.^14^

Symptomatology in inverted stress cardiomyopathy is very similar to ACS or ischemic heart disease. It may present with chest pain (> 75%), shortness of breath (> 50%), dizziness (> 25%), syncope (5% to 10%), abdominal pain, and nausea.^8^ Cardiac troponin is frequently elevated, with higher-than-usual levels found in classic stress cardiomyopathy.^15^ ECG can be used as a differentiating factor between ACS and stress cardiomyopathy. ECG changes are present in 95% of the cases, with ST segment elevation, nonspecific T-wave inversion, and QTc prolongation commonly developing 24 to 48 hours after symptom onset.^16^ Diagnostic investigations include blood analysis, ECG, transthoracic echocardiogram, cardiac magnetic resonance imaging, and coronary angiogram. Studies have identified the role of BNP in stress-induced cardiomyopathy.^15,17^ It is a good way to differentiate classic from inverted types, as levels are significantly higher in the former.^15^ BNP rises 24 hours after the onset of symptoms and can remain elevated for 3 months, providing a useful indicator of severity of LV dysfunction through correlation with LVEF.^18^ However, we did not measure these levels. Cardiac troponin levels measured by conventional assays also remain elevated in > 90% of cases but generally < 10 mg/mL, which was observed in our case as well.^8,19^

Management of inverted stress cardiomyopathy is mainly supportive, with treatment guidelines similar to stress cardiomyopathy. The role of guideline-directed medical therapy has demonstrated mixed results in stress cardiomyopathy. Beta blockers must be used at the clinician’s discretion to prevent arrhythmias.^20^ Anticoagulation must be considered only if there is a significant area of akinesis; in the event a thrombus develops, anticoagulation can be given for 3 months.^21^ ACE inhibitors showed a marginal 1-year benefit, and calcium channel blockers showed a shorter recovery period.^8,22^ Dual antiplatelet therapy did not play a role; in fact, it increased mortality.^23^ The recurrence rates and outcomes are similar to classic stress cardiomyopathy, averaging 2% to 4% per year.^24^

## Conclusion

Inverted stress cardiomyopathy and classic stress cardiomyopathy may present similarly but in a different patient population. In our patient, the most likely cause of inverted stress cardiomyopathy was likely attributed to stress in the setting of a surgical procedure. Her age also fits the demographic characteristics for the inverted variant of stress cardiomyopathy. It is important to contemplate stress-induced cardiomyopathies in pre- and post-menopausal women who are exposed to significant physical and mental stressors to differentiate it from ACS.

## Key Points

- Stress cardiomyopathy must be diagnosed and managed in pre-menopausal as well as in postmenopausal women with significant stressors.
- Stress cardiomyopathy must be differentiated based on a thorough history taking, clinical examination and investigations from ACS.
